# Biotransformation of 2,4‐dinitrotoluene in a phototrophic co‐culture of engineered *Synechococcus elongatus* and *Pseudomonas putida*

**DOI:** 10.1111/1751-7915.13544

**Published:** 2020-02-16

**Authors:** Derek T. Fedeson, Pia Saake, Patricia Calero, Pablo Iván Nikel, Daniel C. Ducat

**Affiliations:** ^1^ DOE‐MSU Plant Research Laboratories Michigan State University East Lansing MI USA; ^2^ Genetics Program Michigan State University East Lansing MI USA; ^3^ Heinrich‐Heine Universität Düsseldorf Germany; ^4^ The Novo Nordisk Foundation Center for Biosustainability Technical University of Denmark Kgs Lyngby Denmark; ^5^ Department of Biochemistry and Molecular Biology Michigan State University East Lansing MI USA

## Abstract

In contrast to the current paradigm of using microbial mono‐cultures in most biotechnological applications, increasing efforts are being directed towards engineering mixed‐species consortia to perform functions that are difficult to programme into individual strains. In this work, we developed a synthetic microbial consortium composed of two genetically engineered microbes, a cyanobacterium (*Synechococcus elongatus* PCC 7942) and a heterotrophic bacterium (*Pseudomonas putida* EM173). These microbial species specialize in the co‐culture: cyanobacteria fix CO_2_ through photosynthetic metabolism and secrete sufficient carbohydrates to support the growth and active metabolism of *P. putida*, which has been engineered to consume sucrose and to degrade the environmental pollutant 2,4‐dinitrotoluene (2,4‐DNT). By encapsulating *S. elongatus* within a barium–alginate hydrogel, cyanobacterial cells were protected from the toxic effects of 2,4‐DNT, enhancing the performance of the co‐culture. The synthetic consortium was able to convert 2,4‐DNT with light and CO_2_ as key inputs, and its catalytic performance was stable over time. Furthermore, cycling this synthetic consortium through low nitrogen medium promoted the sucrose‐dependent accumulation of polyhydroxyalkanoate, an added‐value biopolymer, in the engineered *P. putida* strain. Altogether, the synthetic consortium displayed the capacity to remediate the industrial pollutant 2,4‐DNT while simultaneously synthesizing biopolymers using light and CO_2_ as the primary inputs.

## Graphical abstract

This work describes the development of a synthetic microbial consortium composed of a cyanobacterium engineered to use light to fix inorganic carbon, and a heterotrophic bacterium engineered to degrade the environmental pollutant, 2,4‐dinitrotoluene. Together, the synthetic consortium displayed the capacity to remediate the industrial compound 2,4‐DNT while simultaneously synthesizing biopolymers using light and carbon dioxide as the primary inputs.

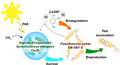

## Introduction

In nature, bacteria typically coexist in communities with hundreds to thousands of species, creating a complex web of interspecies metabolic reactions (Little *et al.*, 2008; Saxena, 2015; Dolinšek *et al.*, 2016; Goldford *et al.*, 2018). Most consortia exhibit a high degree of *division of labour*, where individual species display specialized metabolism and exchange small molecules and signals with neighbours (Tan *et al.*, 2015). Interactions range from the cooperative degradation of toluene and other aromatic compounds (Nikel *et al.*, 2014; Tecon and Or, 2017) to the consumption of metabolic waste products (Wilkinson *et al.*, 1974). Compartmentalization of biochemical transformations across distinct species can confer metabolic capabilities on a consortium that may be difficult to engineer within any one individual species (Roell *et al.*, 2019). Natural microbial consortia also exhibit other desirable features, such as robustness in the face of fluctuating environmental conditions and invasive microbes (Blasche *et al.*, 2017). Against this background, there has been increasing interest in rationally engineering microbial consortia for desired outputs to capitalize upon some of these potential benefits (Ortíz‐Marquez *et al.*, 2013; McCarty and Ledesma‐Amaro, 2019; Tsoi *et al.*, 2019).

Recently, cyanobacteria have been engineered to efficiently produce and secrete simple sugars, making them attractive as alternative feedstock crops to produce carbohydrates for consumption by other industrially relevant microbes (Carrieri *et al.*, 2012; Xu *et al.*, 2013; Hays and Ducat, 2015; Song *et al.*, 2016; Hays *et al.*, 2017; Santos‐Merino *et al.*, 2019; Vijay *et al.*, 2019). In one such example, the heterologous expression of a sucrose permease gene (*cscB*) in *Synechococcus elongatus* PCC 7942 (hereafter, *S. elongatus* CscB) enabled light‐driven secretion of sucrose at rates estimated to exceed the productivities of traditional plant feedstock crops (Ducat *et al.*, 2012). One approach to utilize the sugars secreted by such cyanobacteria is to cultivate them with a heterotrophic partner that can convert the carbohydrate into value‐added products. Recent reports have shown that *S. elongatus* CscB can be paired with a variety of heterotrophic microbes, including *Escherichia coli* and *Bacillus subtili*s (Hays *et al.*, 2017), *Azotobacter vinelandii* (Smith and Francis, 2016, 2017), *Halomonas boliviensis* (Weiss *et al.*, 2017), *Pseudomonas putida* (Löwe *et al.*, 2017a), *Saccharomyces cerevisiae* (Ducat *et al.*, 2012) and *Cryptococcus curvatus* and *Rhodotorula glutinis* (Li *et al.*, 2017). Such co‐cultures are typically stable over long time periods (weeks to months) and the heterotrophic partner strain can be programmed to output value‐added biological products, such as α‐amylase (Hays *et al.*, 2017), fatty acids (Li *et al.*, 2017) and polyhydroxyalkanoates (PHAs) [e.g. poly(3‐hydroxybutyrate) (Smith and Francis, 2016; Smith and Francis, 2017; Weiss *et al.*, 2017; Löwe *et al.*, 2017a,b)].

The limited availability of potable water influences the feasibility of algal and cyanobacterial applications; cultivation using marginal water supplies may ultimately be more sustainable (Barry *et al.*, 2016). Industrial wastewater streams are often unsuitable for plant‐based agriculture because of the toxic contaminants they contain. 2,4‐Dinitrotoluene (2,4‐DNT) is a nitroaromatic xenobiotic that is produced as a by‐product during the synthesis of polyurethane, pesticides and explosives (Spain, 1995; Ju and Parales, 2010). A single 2,4,6‐trinitrotoluene (TNT)‐manufacturing plant can contaminate up to 500 000 gallons of water with nitroaromatics in a single day (Yinon, 1990) – and 2,4‐DNT is a significant toxic contaminant found at dangerous levels in water and soils (Hooker and Skeen, 1999). 2,4‐DNT is highly stable within the environment and remediation costs *via* incineration have been estimated at 400 USD per cubic yard of soil (Griest *et al.*, 1995, 1998).

An alternative strategy for remediation of sites contaminated with 2,4‐DNT is the use of bacterial strains capable of mineralizing this toxic nitroaromatic (Symons and Bruce, 2006; Aburto‐Medina *et al.*, 2017). Generally, two biological pathways that transform 2,4‐DNT are found in nature. The non‐specific reductive pathway is thought to involve reduction of 2,4‐DNT by common electron carriers such as flavin‐ or iron center‐containing electron donors (e.g. nitroreductases; French *et al.*, 2001; Shemer *et al.*, 2018). Reduction of the nitro group of 2,4‐DNT creates highly reactive nitroso‐intermediates and hydroxylamino‐derivatives, which are also toxic due to their capacity to bind a range of biomolecules, frequently causing cellular damage by forming DNA and protein adducts (Spain, 1995; Achtnich *et al.*, 1999; Padda *et al.*, 2003). The toxic derivatives formed by the reductive pathway are not fully mineralized by most bacteria and also have long persistence times in natural environments (Achtnich *et al.*, 1999; Shemer *et al.*, 2018). A separate pathway that initiates with 2,4‐DNT oxidation has been identified in some prokaryotes, including *Burkholderia* sp. R34 (McCormick *et al.*, 1978; Spanggord *et al.*, 1991; Suen *et al.*, 1996), where it is encoded in a single gene cluster (Suen and Spain, 1993; Haigler *et al.*, 1994; Parales *et al.*, 1996; Johnson *et al.*, 2000; Nishino *et al.*, 2000). Enzymes encoded by the *dnt* gene cluster catalyse the oxidation of 2,4‐DNT through a series of steps ultimately resulting in the release of nitrite, pyruvate and propionyl‐coenzyme A (CoA) (Johnson *et al.*, 2002). Because the products of the oxidative pathway are non‐toxic metabolites common to most microorganisms, the oxidative pathway is preferable from the perspective of bioremediation.

**Figure 1:**
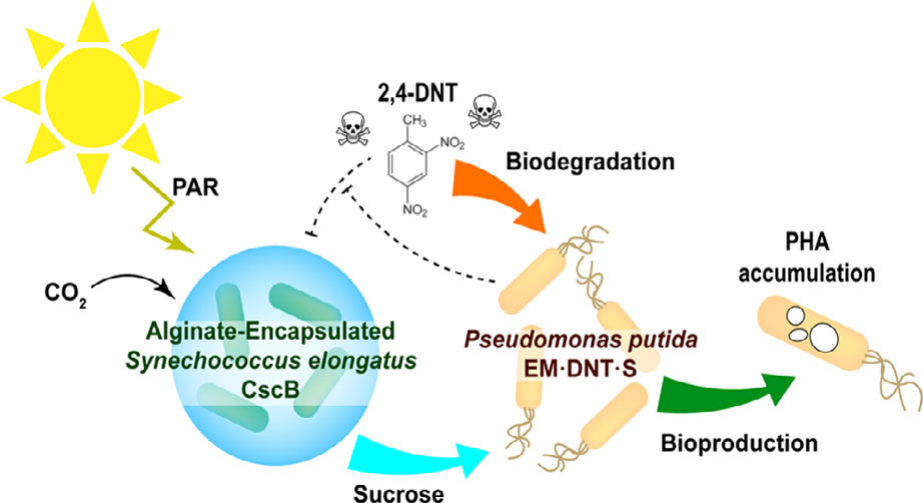
Photosynthetic co‐culture of engineered *Synechococcus elongatus* and *Pseudomonas putida* designed for simultaneous biodegradation and bioproduction. *S*. *elongatus* CscB, encapsulated within alginate beads, fix carbon dioxide *via* the Calvin‐Benson‐Bassham cycle driven by photosynthetically active radiation (PAR). The fixed carbon is converted into sucrose and exported through a heterologous sucrose permease (CscB) into the culture medium. The exported sucrose is consumed by *P. putida* EM·DNT·S for growth, supporting biotransformation of the toxic pollutant 2,4‐dinitrotoluene (2,4‐DNT), and concurrent production of polyhydroxyalkanoate (PHA).

In this study, we expanded upon prior efforts of engineering synthetic cyanobacteria/heterotroph consortia (Hays and Ducat, 2015; Smith and Francis, 2016, 2017; Hays *et al.*, 2017; Weiss *et al.*, 2017; Li *et al.*, 2017; Löwe *et al.*, 2017a) with the goal of generating co‐cultures that can utilize contaminated water supplies and perform a bioremediation function while also producing a valuable bioproduct from light and CO_2_ (Fig. 1). We desired a heterotrophic partner that was genetically tractable and which could tolerate the toxic effects of 2,4‐DNT – therefore, we turned our attention to the soil bacterium *P*. *putida*, an emerging microbial *chassis* (Calero and Nikel, 2019) recognized for its high tolerance to aromatic organic compounds, versatile central metabolism and genetic tractability (Park *et al*., 2006; Nikel *et al.*, 2016; Nikel and de Lorenzo, 2018; Wirth *et al.*, 2020). We explored the capacity of *S. elongatus* CscB to support engineered strains of *P. putida* in direct co‐culture without supplementation of other organic carbon sources. Furthermore, *P. putida* was engineered to express the oxidative 2,4‐DNT degradation pathway and the capacity of these synthetic co‐cultures to remove this common toxin from the stream was evaluated both in batch and repeated‐batch cultures. Finally, we explored the capability of these engineered co‐cultures to produce PHA as an accessory function.

## Results

### **Alginate‐encapsulated *S*** *.* ***elongatus* CscB cells are protected from the toxic effects of 2,4‐DNT**

**Figure 2:**
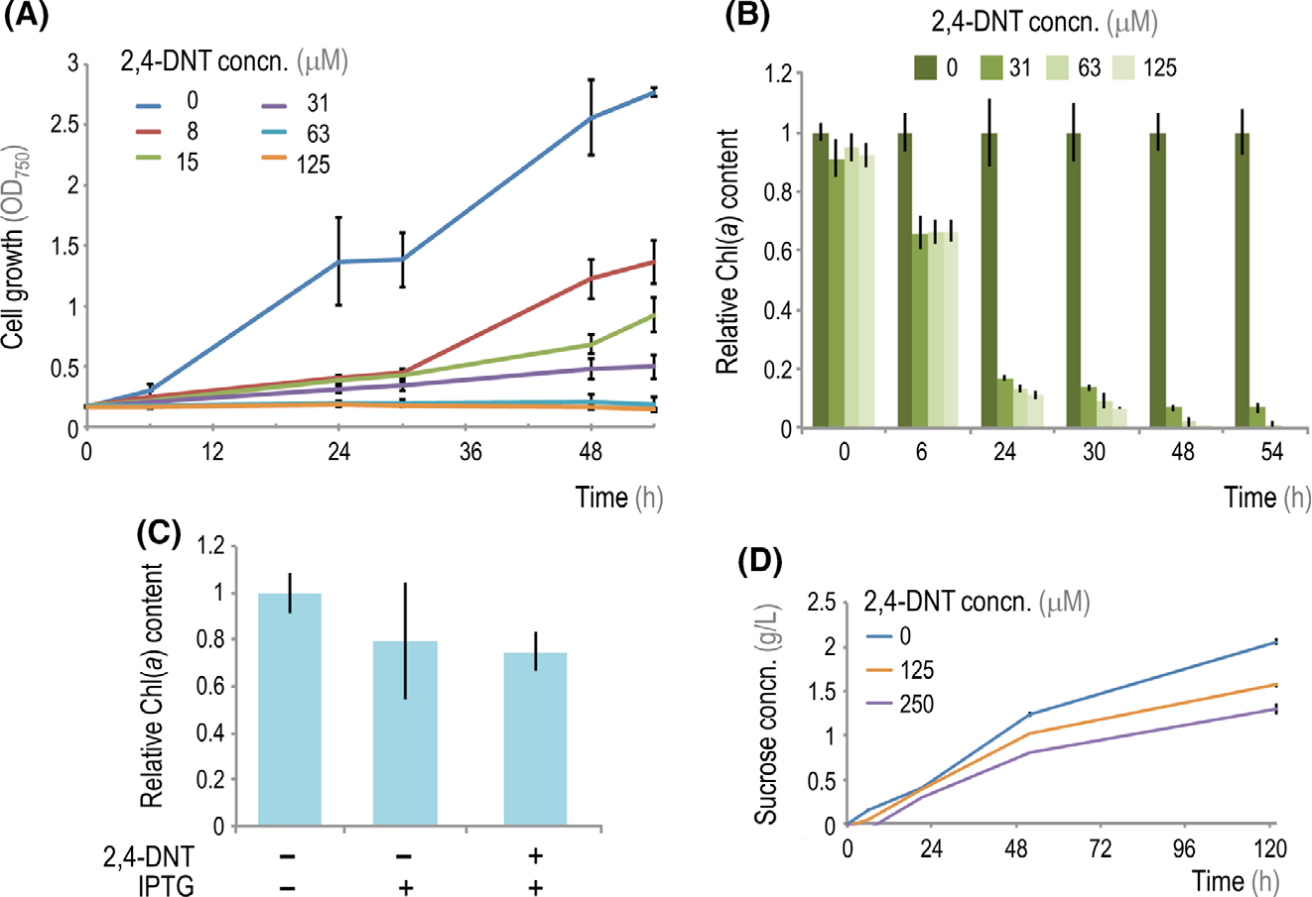
Toxic effects of 2,4‐DNT on planktonic and alginate‐encapsulated *S*. *elongatus* CscB. A. Effect of increasing 2,4‐DNT concentrations (concn.) on the growth of *S*. *elongatus* CscB in planktonic cultures, assessed as the optical density measured at 750 nm (OD_750_). B. The chlorophyll *a* [Chl(*a*)] content in cells of the cultures shown in panel (A) was measured and normalized to that in control cultures with no added 2,4‐DNT. C. Chl(*a*) content of alginate‐encapsulated *S*. *elongatus* CscB cells after 5 days of exposure to 2,4‐DNT (250 μM) in M3 minimal medium with or without IPTG (1 mM) as indicated. Chl(*a*) content values were normalized to that in control cultures without any additives. D. Total sucrose concentration in the culture supernatant of induced (*via* addition of 1 mM IPTG) cultures of alginate‐encapsulated *S. elongatus* CscB grown in the presence of different 2,4‐DNT concentrations. In panels (A–C), the mean values for *n* = 3 are displayed, and error bars represent standard deviations; in panel (D), the mean values for *n* = 3 with three technical replicates are shown, and error bars represent standard deviations.

2,4‐DNT is broadly toxic to many prokaryotes and eukaryotes (Yoon *et al.*, 2006; Kuperman *et al.*, 2009; Rocheleau *et al.*, 2010); therefore, we first characterized the susceptibility of *S. elongatus* to this nitroaromatic compound at concentrations relevant for subsequent co‐culture applications. We observed that low concentrations of 2,4‐DNT (8 μM) started to cause a substantial (ca. 60%) growth impairment in planktonic cultures of *S. elongatus*, while growth was arrested at concentrations ≥ 15 μM (Fig. 2A). Likewise, the content of chlorophyll *a* [Chl(*a*)], the loss of which is an indicator of physiological stress and cell death (Sauer *et al.*, 2001; Latifi *et al.*, 2009; Korosh *et al.*, 2018), declined rapidly in *S. elongatus* cells exposed to 2,4‐DNT concentrations above 30 μM (Fig. 2B and Fig. S1). In particular, we could detect a decrease of ca. 40% in the relative Chl(*a*) content over the first 6 h of incubation – and visible bleaching of cultures occurred by 48 h at 2,4‐DNT concentrations ≥ 30 μM. By comparison, soils and water from contaminated sites are routinely reported to have 2,4‐DNT levels near or above 100 μM (Griest *et al.*, 1995). These preliminary experiments indicated that low 2,4‐DNT levels could significantly affect the growth and physiological status of planktonic *S. elongatus* cells, a complication that could preclude their use to support bioremediation of this compound in co‐cultures.

Previously, we have shown that *S. elongatus* cells encapsulated within an alginate hydrogel remain metabolically active for months under prolonged nitrogen‐depletion stress (Weiss *et al.*, 2017). Similarly, immobilization of a variety of cell types in hydrogels has been reported to increase stress tolerance and cell longevity (Bailliez *et al.*, 1985; Gilleta *et al.*, 2000; Leino *et al.*, 2012; Therien *et al.*, 2014; Ruiz‐Güereca and Sánchez‐Saavedra, 2016). We therefore examined the viability of alginate‐encapsulated *S. elongatus* CscB cells and found that they maintained relative Chl(*a*) contents at a level similar to that of planktonic cells, even when exposed to 2,4‐DNT concentrations near the solubility limits (250 μM) (Fig. 2C and Fig. S2 and S3). As indicated in Fig. S3B, the Chl(*a*) content per cell in alginate‐encapsulated *S. elongatus* CscB was ca. 1 × 10^–8^ μg cell^−1^, irrespective of the addition of 250 μM 2,4‐DNT and 1 mM IPTG (the inducer needed to stimulate sucrose excretion by means of the CscB exporter). Furthermore, these encapsulated *S. elongatus* CscB cells also retained metabolic activity for at least 5 days when exposed to 250 μM 2,4‐DNT – as assayed by the sucrose secreted to the supernatants of IPTG‐induced cultures (Fig. 2D). In particular, the sucrose concentration in supernatants of cultures added with 250 μM 2,4‐DNT reached ca. 1.2 g l^−1^ by the end of the experiment. Therefore, these experiments indicate that alginate encapsulation of *S. elongatus* CscB sufficiently protects cells from the acute toxicity brought about by 2,4‐DNT addition without affecting the capacity of the cells to produce and secrete sugars, which led us to explore co‐cultivation strategies as indicated below.

### **Co‐cultivation of engineered derivatives of *P*** *.* ***putida* EM173 with alginate‐encapsulated *S*** *.* ***elongatus* CscB**

**Figure 3:**
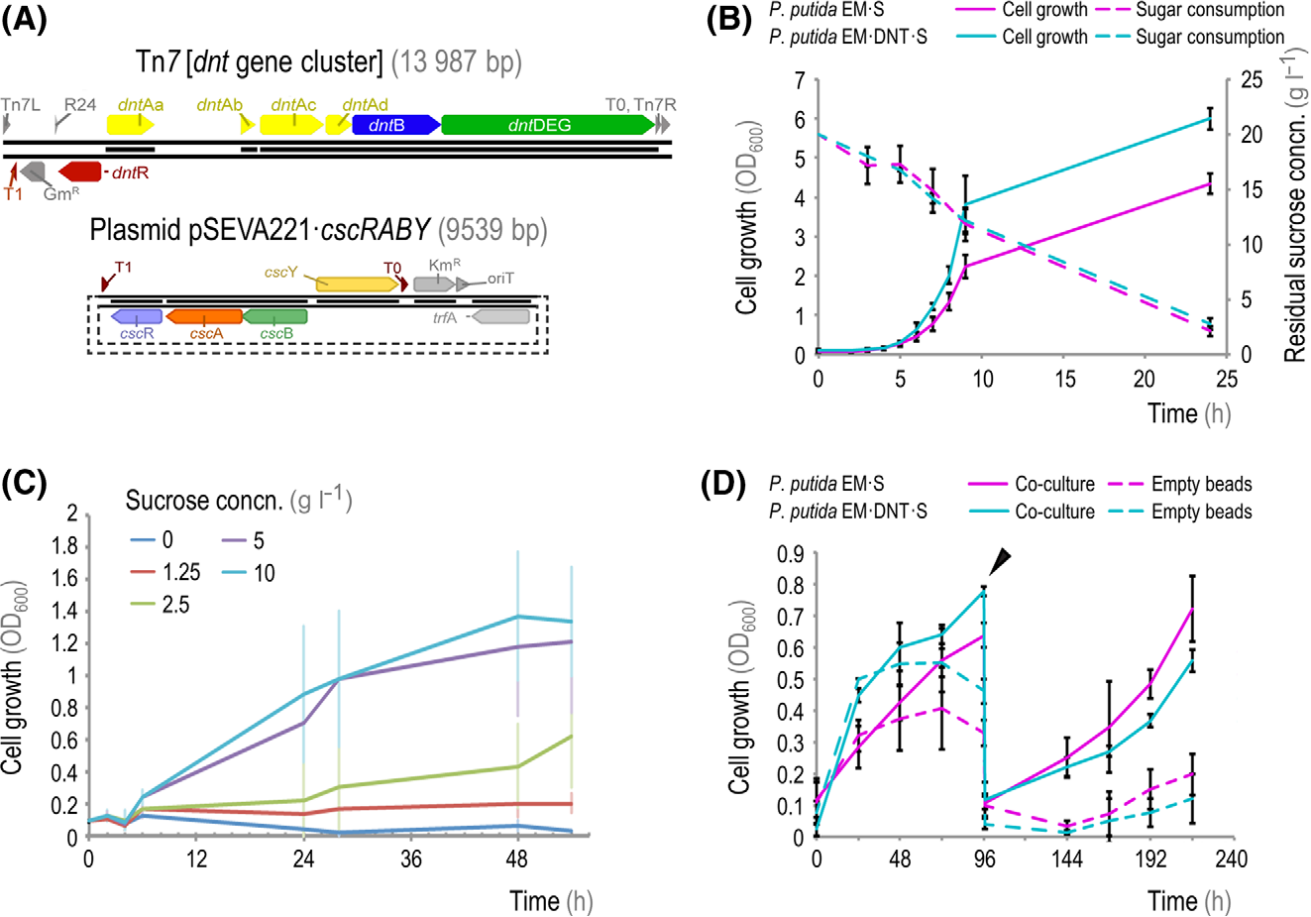
Growth characterization of *P. putida* strains engineered for sucrose consumption and 2,4‐DNT transformation. A. Schematic representation of the gene cluster encoding the enzymes involved in 2,4‐DNT degradation from *Burkholderia* sp. R34, transferred into the *P*. *putida* chromosome *via* Tn*7* transposition (*top panel*), and plasmid pSEVA221·*cscRABY* (Löwe *et al.*, 2019), encoding the functions needed for sucrose utilization from *P*. *protegens* Pf‐5 (*bottom panel*). B. Growth and sucrose utilization by engineered *P. putida* strains in M9 minimal medium containing 20 g l^−1^ sucrose. Bacterial growth was assessed as the optical density measured at 600 nm (OD_600_); residual sucrose concentration (concn.) in culture supernatants was determined with an enzymatic assay. (C) Growth of *P. putida* EM·DNT·S in M3 minimal medium containing different concentrations of sucrose (0, 1.25, 2.5, 5 and 10 g l^−1^). D. The growth of *P. putida* EM·S and *P. putida* EM·DNT·S, inoculated from overnight cultures with 20 g l^−1^ sucrose, was monitored with or without encapsulated *S. elongatus* CscB (indicated as *co‐culture*). OD_600_ measurements track only the growth of the *P. putida* cells (i.e. planktonic cells). At 96 h post‐inoculation (slanted arrowhead), all of the M3 minimal medium was removed and replaced with fresh medium, allowing the residual *P. putida* cells on the surface of the alginate beads to repopulate the culture. In panels (B) and (C), the mean values for *n* = 4 are displayed, and error bars represent standard deviations. In panel (D), the mean values for *n *> 3 are displayed from experiments carried in different days, and error bars represent standard deviations.

We next constructed a strain of *P. putida* that is capable of (i) utilizing sucrose as a carbon source and (ii) transforming 2,4‐DNT through the oxidative pathway from *Burkholderia* sp. R34. *P. putida* does not naturally utilize sucrose as a carbon source, but has been previously engineered to grow on this substrate (Löwe *et al.*, 2017a,b). We first transformed the genetically tractable, prophage‐less strain EM173 of *P. putida* (Martínez‐García *et al.*, 2015) with the low‐copy‐number plasmid pSEVA221·*cscRABY* (Löwe *et al.*, 2019) that bears the sucrose‐utilization genes from *P. protegens* Pf‐5 (Fig. 3A). The resulting strain was named *P. putida* EM·S. Plasmid pSEVA221·*cscRABY*, a derivative of vector pSEVA221 (Silva‐Rocha *et al.*, 2013), constitutively expresses the genes encoding a sucrose hydrolase (CscA, PFL_3237) and a sucrose permease (CscB, PFL_3238). These functions enable the uptake and catabolism of the disaccharide, together with a cognate transcriptional regulator (CscR, PFL_3236). Separately, a synthetic mini‐Tn*7* transposon, carrying the functions required for 2,4‐DNT biotransformation from *Burkholderia* sp. R34 (Fig. 3A), was constructed as indicated in *Experimental Procedures*. The *dnt* gene cluster in this transposon was delivered into the unique *att*·Tn*7* site within the chromosome of *P. putida* EM·S, resulting in a stable, engineered strain designed for both oxidative 2,4‐DNT transformation and sucrose consumption (hereafter referred to as *P. putida* EM·DNT·S).

We then examined the sucrose catabolism capacity of these modified *P. putida* strains under a range of physiologically relevant growth conditions. We observed that the pSEVA221·*cscRABY* plasmid was sufficient to confer sucrose utilization in both *P. putida* EM·S and *P. putida* EM·DNT·S when grown in M9 minimal medium supplemented with 20 g l^−1^ sucrose (Fig. 3B). Both *P. putida* EM·S and *P. putida* EM·DNT·S grew exponentially over the course of the first 9 h post‐inoculation, and by 24 h had reached final cell densities of OD_600_ (optical density measured at 600 nm) of 4.3 and 6 respectively. The specific growth rates of the two engineered strains were very similar, and the pattern of sucrose consumption was almost identical in either case. Indeed, during this incubation period, sucrose concentration declined in a nearly linear fashion from 20 to < 3 g l^−1^ at 24 h (Fig. 3B). We similarly evaluated the growth profile of engineered *P. putida* at lower sucrose concentrations within M3 minimal medium, designed for cyanobacterial growth (derived from BG‐11 minimal medium; see Table S1). To this end, *P. putida* EM·DNT·S was first inoculated in M3 minimal medium without supplementing a carbon source and incubated overnight in order to deplete internal carbon stores, then washed and re‐inoculated into fresh M3 minimal medium supplemented with a range of sucrose concentrations (from 0 to 10 g l^−1^). Bacterial growth was evident at sucrose concentrations ranging from 1.25 to 10 g l^−1^, after a lag phase longer than that observed in M9 minimal medium (Fig. 3C). In particular, we observed that at sugar concentrations > 5 g l^−1^ the bacterial cultures attained OD_600_ values well over 1 unit after 48 h of incubation. Taken together, these results indicate that this cyanobacterial culture medium supports growth of engineered *P*. *putida* strains when sucrose is present as the sole carbon source – thereby enabling its use for designing co‐cultures.

Next, we implemented and evaluated co‐cultures by inoculating *P. putida* EM·S and *P. putida* EM·DNT·S into flasks containing either empty alginate beads or alginate‐encapsulated *S. elongatus* CscB (Fig. 3D). Within the first 48 h, *P. putida* cells grew in all flasks – even in those containing empty alginate beads. Residual carbon stores within *P. putida* and/or trace amounts of sugars present in the alginate beads likely drove the residual bacterial growth observed in flasks containing empty beads, and the growth of these cultures was minor compared to that of cells co‐cultivated with alginate‐encapsulated (and sucrose‐producing) *S. elongatus* CscB (Fig. 3D). Furthermore, when the alginate beads were re‐suspended in fresh M3 minimal medium at 96 h post‐inoculation, growth of engineered *P. putida* strains was only observed in flasks with alginate‐encapsulated *S. elongatus* CscB cells, where they grew exponentially. Additionally, we did not observe degradation of the alginate beads in these (nor in subsequent) experiments, indicating that *P. putida* cannot utilize the hydrogel itself as a carbon source, in agreement with previous work (Löwe *et al.*, 2017a). With these results at hand, we next evaluated the use of this co‐culture setup for 2,4‐DNT transformation.

### **Biotransformation of 2,4‐DNT by *P*** *.* ***putida* EM·DNT·S**

**Figure 4:**
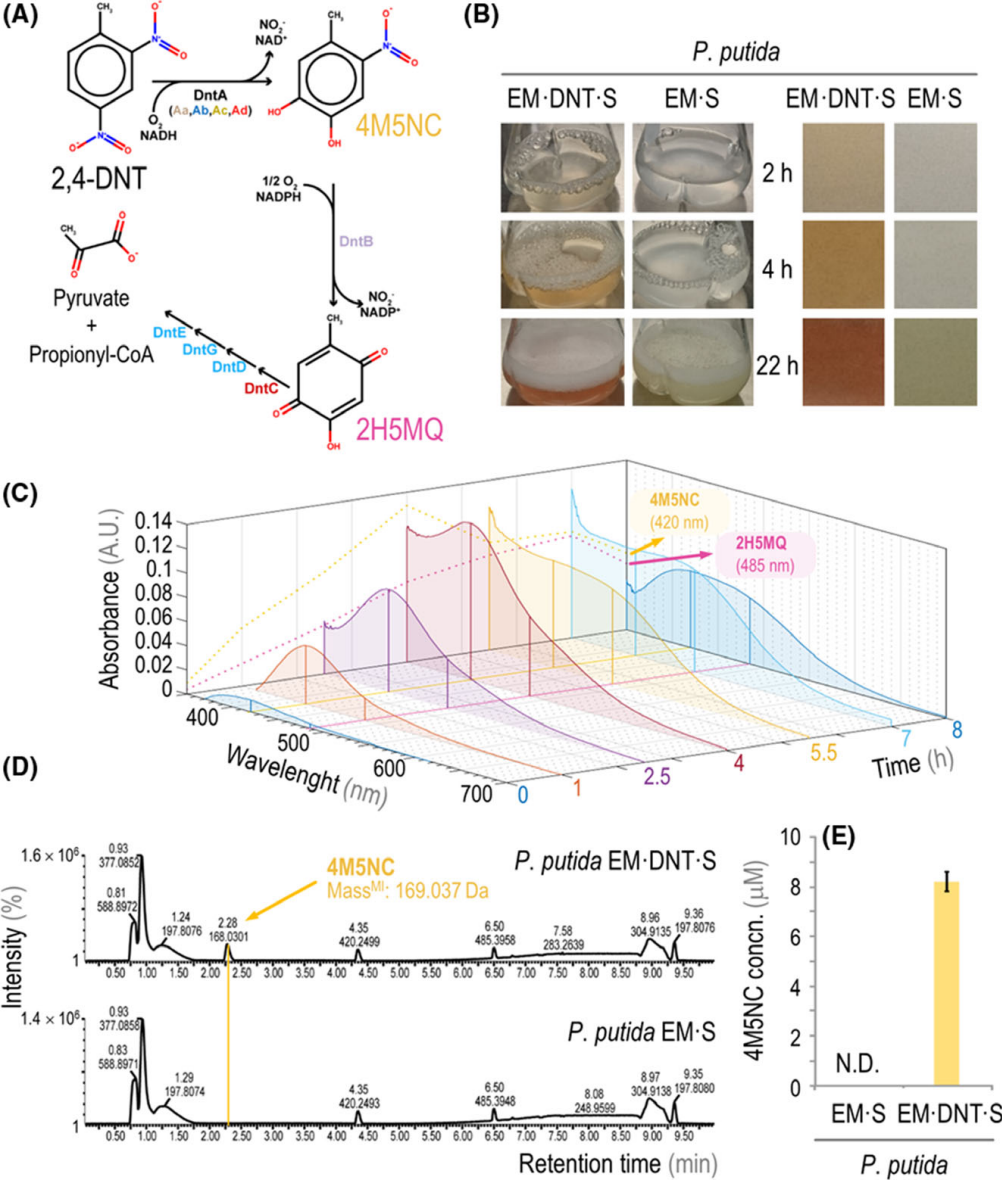
2,4‐DNT biotransformation in mono‐cultures of engineered *P. putida*. A. Pathway for the oxidative degradation of 2,4‐DNT in *Burkholderia* sp. R34. The first step, executed by DntA, is a di‐oxygenation reaction that leads to the formation of 4‐methyl‐5‐nitrocatechol (4M5NC). This intermediate is the substrate for the DntB mono‐oxygenase, yielding 2‐hydroxy‐5‐methylquinone (2H5MQ). In the native context, the pathway leads to the conversion of 2,4‐DNT into pyruvate and propionyl‐coenzyme A (CoA). B. Representative images of axenic cultures of *P. putida* EM·DNT·S and *P. putida* EM·S grown in the presence of 250 μM 2,4‐DNT over time (*left column*); the zoom‐in of supernatants from these cultures shows the characteristic changes in pigmentation (*right column*). C. Time‐course of averaged spectral signatures (*n* = 3) in supernatants from *P. putida* EM·DNT·S cultures grown in the presence of 250 μM 2,4‐DNT, as measured *via* scanning spectrophotometry. The signature absorbance peaks of 4M5NC and 2H5MQ are indicated. A.U., arbitrary units. D. LC‐MS analysis of culture supernatants of *P. putida* EM·DNT·S (*top panel*) and *P. putida* EM·S (*bottom panel*) after 4 h of incubation with 250 μM 2,4‐DNT. The peak corresponding to the monoisotopic mass of 4M5NC (mass^MI^) is indicated by a yellow line. E. LC‐MS quantification of the 4M5NC concentration (concn.) in supernatants of *P. putida* EM·S and *P. putida* EM·DNT·S cultures after 4 h incubation with 250 μM 2,4‐DNT. All cultures were grown in M3 minimal medium with sucrose at either 20 g l^−1^ (B) or 2 g l^−1^ (C–E). N.D., not detected.

As indicated previously, the *dnt* gene cluster of *Burkhoderia* sp. R34 encodes the enzymes needed for oxidative breakdown of 2,4‐DNT through oxygenation reactions (Spain, 1995; Symons and Bruce, 2006). The enzymes within the 2,4‐DNT degradation pathway catalyse two successive oxygenation reactions mediated by DntA and DntB (Fig. 4A). 2,4‐DNT is firstly dioxygenated by DntA to form 4‐methyl‐5‐nitrocatechol (4M5NC), a compound with a strong absorption peak at 420 nm (de las Heras *et al.*, 2011). 4M5NC is the substrate of DntB, which yields 2‐hydroxy‐5‐methylquinone (2H5MQ), an intermediate with an absorption peak at 485 nm (de las Heras *et al.*, 2011). In the native context of this catabolic route, 2H5MQ is then processed by the products of a number of additional genes encoded in the pathway to finally yield pyruvate and propionyl‐CoA (Fig. 4A).

We next examined the functional capacity of the *dnt* gene cluster introduced into *P. putida* EM·DNT·S by following the kinetics of 2,4‐DNT transformation in axenic cultures. M3 minimal medium, supplemented with 250 μM 2,4‐DNT and 2 g l^−1^ sucrose, was inoculated with *P. putida* EM·S or *P. putida* EM·DNT·S at an initial OD_600_ = 0.1. Consistent with the sequential formation of two expected oxidative intermediates, the supernatant of the *P. putida* EM·DNT·S cultures visibly changed pigmentation over the course of the growth experiment (Fig. 4B), whereas cultures of *P. putida* EM·S did not show any visible changes in coloration over the entire incubation period. Within 2–4 h following inoculation of *P. putida* EM·DNT·S, the supernatant turned yellow, consistent with the accumulation of the first intermediate, 4M5NC. Subsequently, the supernatant transitioned from yellow to orange (around 4 h) and then to red (22 h after inoculation), which reveals the presence of the second intermediate, 2H5MQ, and the breakdown of 4M5NC. These results are indicative of the biotransformation of 2,4‐DNT and, as reported previously, *P*. *putida* strains carrying the *dnt* gene cluster were not able to grow on 2,4‐DNT as the only carbon source (Akkaya *et al.*, 2018). A separate quantitative analysis of the absorbance spectra of *P. putida* EM·DNT·S supernatants focused upon the first 8 h following 2,4‐DNT addition, revealing a characteristic absorbance rise at 420 nm (between 1‐4 h post‐inoculation). A second absorption peak arose at 485 nm (4–8 h), during the time period where the characteristic peak of 4M5NC gradually decreased (Fig. 4C). These spectroscopic changes were neither observed in control experiments with the non‐degrading *P. putida* EM·S strain (Fig. S4) nor in the absence of 2,4‐DNT. We directly confirmed the accumulation of 4M5NC by liquid chromatography coupled to mass spectrometry (LC‐MS). *P. putida* EM·DNT·S supernatants exhibited a peak matching the 4M5NC standard (Fig. 4D), whereas no 4M5NC was detected in control (*P. putida* EM·S) cultures under any condition. Direct quantification of 4M5NC levels by LC‐MS indicated a peak consistent with 4M5NC accumulation (ca. 8 μM) at 4 h post‐inoculation (Fig. 4E). Again, we could not detect any 4M5NC formation in control cultures of the non‐degrading strain *P. putida* EM·S. As an additional control of the activity of the first enzymes within the pathway, we carried experiments in which the release of nitrite from the substrate was measured by a colorimetric method. Nitrite could be detected in culture supernatants when *P. putida* EM·DNT·S was incubated in the presence of 250 μM 2,4‐DNT and using sucrose at 2 g l^−1^ as the sole carbon source. After 4 h, the nitrite concentration reached 7.3 ± 0.2 μM, and it peaked at 59.8 ± 1.3 μM by 24 h, suggesting that the oxidative pathway for 2,4‐DNT degradation plays a significant role in the removal of the nitroaromatic. The decrease in the 4M5NC together with the release of nitrite are indicative of 2,4‐DNT biotransformation. As previously mentioned, no bacterial growth was detected when *P. putida* EM·DNT·S cells were incubated in the mineral medium containing 2,4‐DNT but no added carbon source – which indicates that this engineered strain can transform the substrate without net assimilation of carbon (or at least not to the extent that would support growth on 2,4‐DNT).

The disappearance of 2,4‐DNT from the supernatant of *P. putida* cultures was directly assessed by gas chromatography coupled to mass spectrometry (GC‐MS) in all the experiments. We observed that the soluble fraction of 2,4‐DNT declined significantly within the first 4 h, and dropped to undetectable levels by 22 h. This kinetic pattern of 2,4‐DNT transformation is similar to what has been reported for *Burkholderia* sp. R34 (Pérez‐Pantoja *et al.*, 2013) and other engineered *P. putida* strains (Akkaya *et al.*, 2018, 2019). Soluble levels of 2,4‐DNT declined in cultures of *P. putida* EM·S as well (Fig. S5A), whereas the concentration of the nitroaromatic remained constant in control flasks that were not inoculated with *P. putida* cells (Fig. S5B). These observations can be accounted for by the fact that a small fraction of 2,4‐DNT became most likely adsorbed (i.e. physically associated) to cell surfaces, a known property of nitroaromatic compounds (Achtnich *et al.*, 1999). Additionally, the reductive pathway (catalysed by non‐specific nitroreductases, which have been reported to be present in many bacterial species) could result in the conversion of 2,4‐DNT into other intermediates (Fig. S5C). To investigate this possibility, we assessed culture supernatants for the presence of 2‐amino‐4‐nitrotoluene/4‐amino‐2‐nitrotoluene (2A4NT/4A2NT), two key isomer intermediates of the reductive pathway, by LC‐MS. These two compounds were detected at low levels (< 2.5 μM) in the supernatant of cultures of both *P. putida* EM·DNT·S and *P. putida* EM·S, but the formation of 2A4NT/4A2NT was significantly lower in cultures of *P. putida* EM·DNT·S (Fig. S5D). This result suggests that the oxidative route competes with the non‐specific reductive pathway in removing 2,4‐DNT in engineered *P*. *putida* strains, with a predominant role of the former over the later in *P. putida* EM·DNT·S. With this information at hand, we set to establish co‐cultures of engineered *P*. *putida* and *S. elongatus* as indicated in the next section.

### **Biotransformation of 2,4‐DNT by a synthetic consortium of engineered *P*** *.* ***putida* and *S*** *.* ***elongatus* CscB**

**Figure 5:**
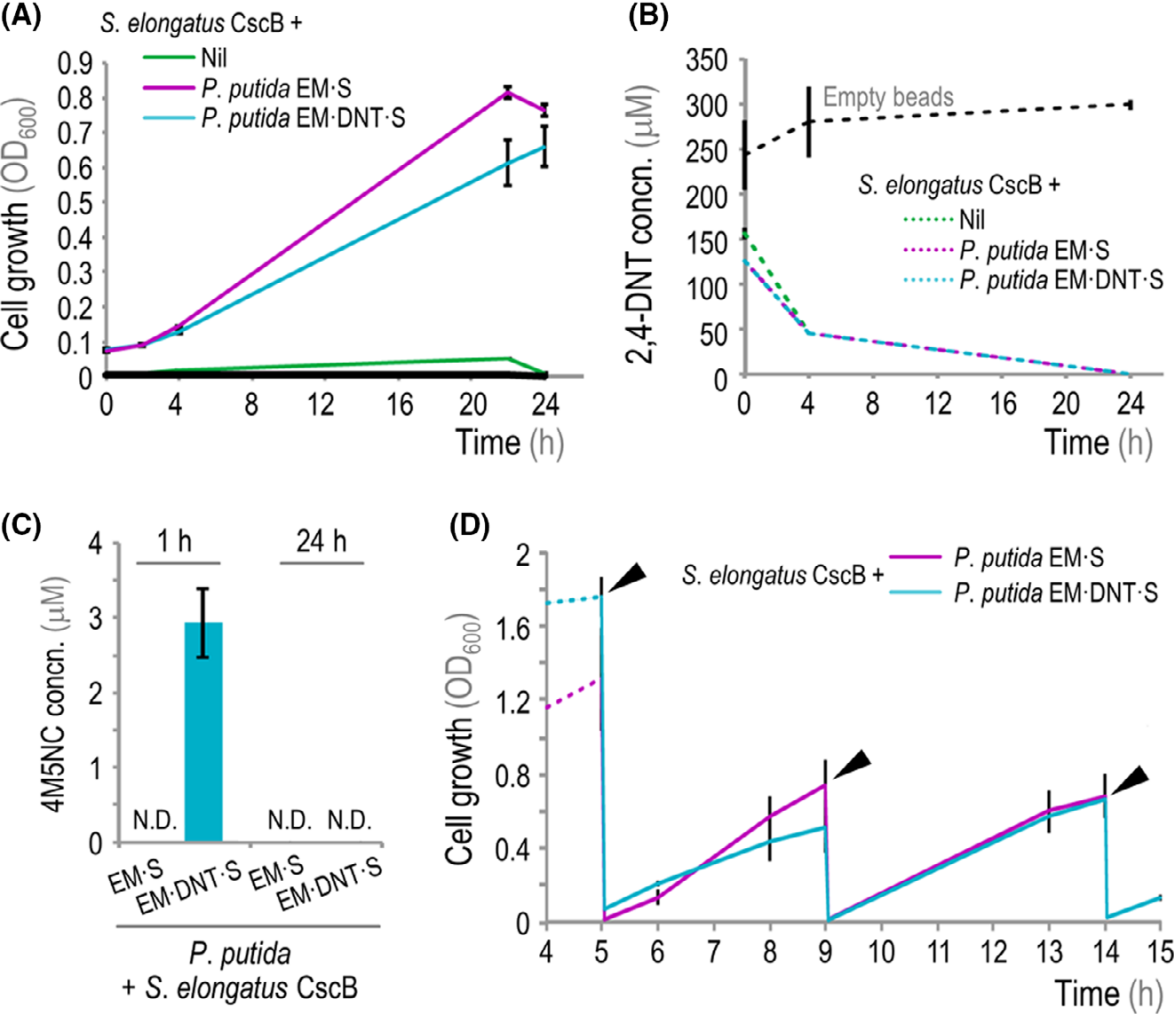
Biotransformation of 2,4‐DNT in co‐cultures of engineered *S. elongatus* and *P. putida*. A. Growth of engineered *P. putida* strains [assessed as the optical density measured at 600 nm (OD_600_)] in co‐culture with alginate‐encapsulated *S. elongatus* CscB in the presence of 250 μM 2,4‐DNT. The black line indicates control experiments with empty beads. B. GC‐MS analysis of 2,4‐DNT concentration (concn.) in supernatants of co‐cultures during 24 h. 2,4‐DNT concentrations dropped below detectable levels by 24 h post‐inoculation. C. LC‐MS quantification of 4M5NC concentration (concn.) in supernatants of co‐cultures after addition of 2,4‐DNT at 250 μM at 1 and 24 h post‐inoculation. N.D., not detected. D. Growth of engineered *P. putida* strains in long‐term co‐cultures with encapsulated *S. elongatus* CscB. The culture medium was exchanged at each indicated time‐point (slanted arroheads), and IPTG and 2,4‐DNT were added at 1 and 250 μM respectively. In panels (A–C), the mean values for *n* = 3 are displayed, and error bars represent standard deviations. In panel (D), the mean values for *n* = 14 are indicated, and error bars represent standard deviations.

We next introduced 2,4‐DNT into co‐cultures of *P. putida* with alginate‐encapsulated *S. elongatus* CscB cells. Both *P. putida* EM·S and *P. putida* EM·DNT·S were able to grow with the beaded *S. elongatus* CscB cells in the presence of 250 μM 2,4‐DNT (Fig. 5A). Cultures containing only alginate‐encapsulated *S. elongatus* CscB or empty alginate beads served as controls for both optical density measurements as well as any potential 2,4‐DNT adsorption to abiotic surfaces. As previously observed, no noticeable bacterial growth was detected in the absence of *S. elongatus* CscB in these experiments. GC‐MS analysis of supernatants demonstrated that cultures containing engineered *P. putida* or *S. elongatus* cells removed 2,4‐DNT from the culture, while the 2,4‐DNT concentration did not change in empty‐bead control experiments (Fig. 5B). Similarly, the supernatant of co‐cultures containing *P. putida* EM·DNT·S exhibited visual changes in colour similar to those reported in Fig. 4, indicating activity of the oxidative *dnt* pathway. Accordingly, LC‐MS analysis validated that the oxidative pathway intermediate 4M5NC transiently accumulated in the supernatants of *P. putida* EM·DNT·S–containing co‐cultures (Fig. 5C), reaching a concentration of ca. 3 μM within the first hour of incubation and completely disappearing after 24 h. Conversely, 4M5NC was not detected in *P. putida* EM·S–containing co‐cultures. These results indicate that the synthetic consortium can efficiently remove 2,4‐DNT in a single batch culture.

Based on these results, we next evaluated the capacity of the co‐culture to remediate multiple (i.e. repeated) batches of 2,4‐DNT–containing streams by extending the co‐culture experiment to 15 days and exchanging the culture supernatant every 4–5 days with fresh medium containing 250 μM 2,4‐DNT (Fig. 5D). In each case, *P. putida* was able to quickly re‐grow from residual planktonic cells after removal and replacement of the culture supernatant – a phenomenon likewise observed for *P. putida* EM·S and *P. putida* EM·DNT·S. Again, colorimetric changes in the supernatant were evident only in the *P. putida* EM·DNT·S–containing co‐cultures after each back‐dilution, with added 2,4‐DNT. Direct measurement of the residual nitroaromatic concentration indicates that the total mass of 2,4‐DNT cleared from solution by the *P. putida* EM·DNT·S strain over this period in repeated‐batch co‐cultures amounts to 136 mg l^−1^.

### Simultaneous removal of 2,4‐DNT and accumulation of PHA by engineered strains of *P*. *putida* in a synthetic consortium

**Figure 6:**
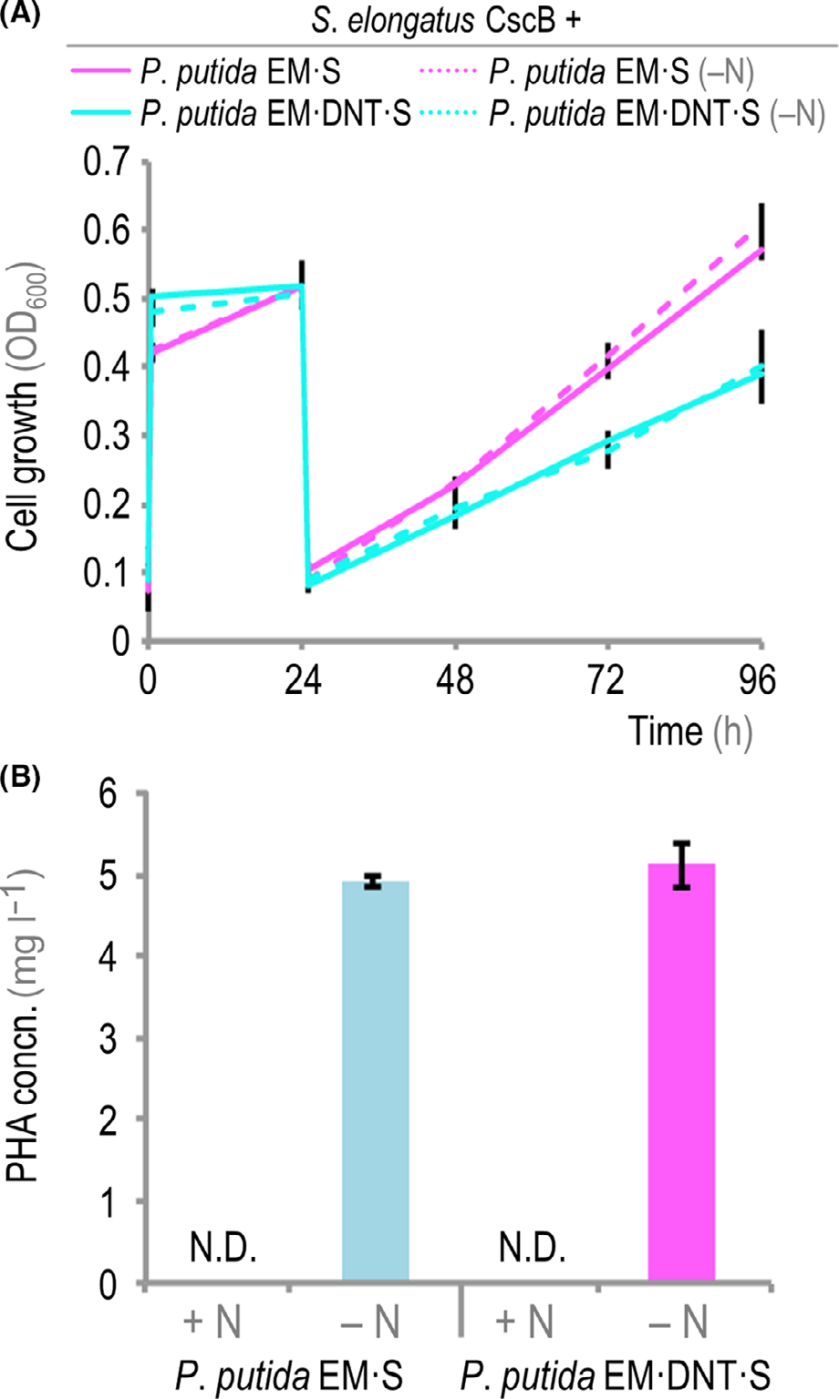
PHA accumulation by engineered *P. putida* in phototrophic co‐cultures. A. Growth of engineered *P. putida* strains in co‐cultures of alginate‐encapsulated *S*. *elongatus* CscB in the presence of 250 μM 2,4‐DNT with or without nitrogen deprivation. Bacterial growth was assessed as the optical density measured at 600 nm (OD_600_). Separate experiments were run in M3 minimal medium with nitrate at the standard concentration (17.6 mM, indicated as + N) or at a reduced concentration (2 mM, indicated as –N; minimal M3‐N medium). B. Quantification of the PHA concentration (concn.) in co‐cultures of engineered *P. putida* strains and alginate‐encapsulated *S*. *elongatus* CscB in the presence of 250 μM 2,4‐DNT after 24 h of incubation into nitrogen‐replete or nitrogen‐reduced minimal M3 medium. In all cases, average values from *n* = 3 are presented, and error bars represent standard deviations. N.D., not detected.

Wild‐type *P. putida* strains are known to accumulate PHA as a carbon storage (López *et al.*, 2015; Prieto *et al.*, 2016), particularly under nitrogen‐depleted conditions in the presence of an abundant carbon source (Hoffmann and Rehm, 2004), which prompted us to investigate the opportunity to both remove 2,4‐DNT and simultaneously produce a valuable by‐product. In these experiments, engineered *P. putida* cells were inoculated into the alginate‐encapsulated and IPTG‐induced *S. elongatus* CscB culture flasks at an OD_600_ ~ 0.5 in the presence of 250 μM 2,4‐DNT (Fig. 6A). These flasks contained either M3 or M3‐N minimal medium, the latter of which has a reduced nitrogen content to trigger polymer accumulation (2 mM NaNO_3_; Table S1). The pattern of cell growth was very similar for the two strains under evaluation, and bacterial growth was not significantly affected by the reduced nitrogen conditions in M3‐N minimal medium. Twenty‐four hours after cycling co‐cultures into either nitrogen‐rich or nitrogen‐depleted media (i.e. M3 and M3‐N minimal medium, respectively), planktonic *P. putida* cells were harvested and the intracellular PHA content was evaluated by means of LC‐MS measurements of crotonic acid as indicated in *Experimental Procedures*. Both engineered *P*. *putida* strains were able to accumulate the polymer under nitrogen‐limited conditions – but not in co‐cultures run in nitrogen‐rich M3 minimal medium (Fig. 6B). In particular, *P. putida* EM·S and *P. putida* EM·DNT·S co‐cultures grown under nitrogen‐depleted conditions in M3‐N minimal medium accumulated the polymer at a specific volumetric productivity of 4.9 and 5.1 mg PHA l^−1^ day^−1^ of culture, with a polymer content in biomass of 22 and 23.4 mg of PHA g cell dry weight^‐1^, respectively. These results indicate that the accumulation of PHA from sucrose as the only carbon source by engineered *P*. *putida* is not affected by the presence of 2,4‐DNT (or its transformation products) and that the polymer can be obtained in co‐cultures designed for 2,4‐DNT removal as an added‐value by‐product.

## Discussion

Rational engineering of microbial communities is an emerging area of study that has implications for both applied and fundamental microbiology. Most natural microbial biochemistry occurs in the context of complex microbial consortia that are unlike the mono‐cultures routinely cultivated under controlled laboratory conditions (Jessup *et al.*, 2004). Desirable system‐level features (e.g. robustness) are thought to emerge from simple interactions between individual species [e.g. by the division of metabolic labour (McCann, 2000; Ives and Carpenter, 2007; Volke and Nikel, 2018)], although these phenomena remain poorly understood. Gaining a deeper understanding of the design principles underlying natural microbial ecologies will likely require the application of top‐down, systems‐level analyses (e.g. meta‐*omic* approaches) and the development of flexible bottom‐up platforms that permit the assembly of simple, tractable microbial mixed cultures (Jessup *et al.*, 2005; Momeni *et al.*, 2011; Goers *et al.*, 2014).

In this work, we constructed microbial co‐cultures capable of transforming the industrial toxin 2,4‐DNT by utilizing light and CO_2_ as the primary inputs. Our present contribution builds upon a flexible co‐cultivation system by demonstrating that (engineered) heterotrophs can be incorporated into designer communities in order to programme them for removal of environmental contaminants – thus extending the utility of synthetic consortia beyond the mere coexistence of microbial strains into performing complex biodegradation tasks. We chose 2,4‐DNT as a model toxicant, since this nitroaromatic compound is a chemical contaminant that poses a significant bioremediation challenge due to toxicity and persistence in natural environments. This synthetic consortium described herein consists of engineered strains of *S. elongatus* and *P. putida* designed to divide metabolic labour between the partner species – with *S. elongatus* CscB specializing in photosynthetic processes, generating and secreting soluble sugars sufficient to promote the growth and distinct metabolic activities of co‐cultivated engineered *P. putida*. We demonstrated that co‐cultures were reproducibly stable over a series of back‐dilutions (repeated‐batch cultivation), permitting the removal of soluble 2,4‐DNT in successive batch cultures for an extended period of time.

The selection of the partner heterotroph in our system is not arbitrary. *P. putida* has gained significant attention as a *chassis* for industrially relevant synthetic biology due to its metabolic diversity, high tolerance of oxidative stress, and capacity to conduct complex biochemical reactions involving aromatic substrates (Poblete‐Castro *et al.*, 2017). In this context, the still‐evolving 2,4‐DNT degradation pathway from *Burkholderia* sp. R34 has been reported to lead to accumulation of oxidative stress an increased rate of mutation in its native context, whereas *P. putida* heterologously expressing the *dnt* gene cluster (*P. putida* EM·DNT and derivatives thereof) does not exhibit the same rate of DNA damage and mutation (Akkaya *et al.*, 2019). *P. putida* EM·DNT·S co‐cultures removed 2,4‐DNT from the soluble phase partly *via* this oxidative degradation pathway. However, under the conditions of our co‐cultures, a portion of 2,4‐DNT is also transformed *via* reductive processes (Fig. S5D) and/or gets adsorbed to surfaces. Because multiple products and nitroamino‐adducts are formed downstream of 2,4‐DNT reduction, we could not precisely quantitate the flux through the oxidative pathway with our data, but we do see a ca. 25% reduction in the accumulation of the reductive products 2A4NT and 4A2NT. Fitting our kinetic data of 2,4‐DNT transformation (Fig. 4 and Fig. S5A) to a simple mass‐action model of a branched metabolic pathway suggests that up to 40% of 2,4‐DNT may be fully mineralized via the oxidative pathway (see *Experimental Procedures*). Further engineering efforts may be required to increase the fraction of 2,4‐DNT that is completely catabolized and/or incorporated into biomass, e.g. by improving the affinity of enzymes in the oxidative pathway for 2,4‐DNT (Monti *et al.*, 2005; Ju and Parales, 2010) or by coupling the growth of engineered strains to fully oxidative catabolism of the aromatic substrate (Durante‐Rodríguez *et al.*, 2018; Fernández‐Cabezón *et al.*, 2019).

In tandem with the transformation of 2,4‐DNT, co‐cultures were programmed to synthesize a valuable by‐product in the form of PHA. Co‐cultures of engineered *P. putida* and *S. elongatus* accumulated the polymer with specific volumetric productivities of ca. 5 mg PHA l^−1^ day^−1^ using gaseous CO_2_ as the primary carbon source – which is lower than the productivity reported in recent publications describing bacterial co‐cultures or mono‐cultures of cyanobacteria directly engineered to express PHA‐producing pathways (Löwe *et al.*, 2017a,b). Nevertheless, we have demonstrated that polymer accumulation can occur in *P*. *putida* the presence of 2,4‐DNT and that PHA synthesis could be coupled to the removal of the nitroaromatic. Therefore, PHA produced from these co‐cultures represents a value‐added by‐product – and a first‐case example of polymer accumulation coupled to bioremediation of an environmental pollutant. The economic prospects of the process can be improved by optimizing PHA yields (e.g. by modifying the regulation of the PHA synthesis to render it insensitive to nitrogen availability) or by incorporating pathways leading to higher‐value bioproducts in the system. Further modifications could include the addition of other microbial partners in the consortium, thereby multiplying the range of products that can be obtained in these co‐culture setups. One way or the other, the rational pairing of cyanobacteria and *P*. *putida*, together with the plethora of synthetic biology approaches created to engineer novel properties in whole‐cell biocatalysts, allows for the design of systems with the potential to transform current production platforms.

## Experimental procedures

### Bacterial strains and culture conditions

Planktonic *S*. *elongatus* strains were grown in a Multitron Pro (Infors HT, Annapolis Junction, MD, USA) photobiological shaker at 32°C, 2% (v/v) CO_2_, and a constant illumination of ~ 70 μmol m^−2^ s^−1^ (15 W Grow‐Lux; OSRAM Sylvania Inc., Wilmington, MA, USA) as previously described (Weiss *et al.*, 2017). Selection for the genomically integrated *cscB* module was achieved by addition of 12.5 μg ml^−1^ chloramphenicol to the culture medium. Engineered *P. putida* strains were constructed essentially as reported previously (Martínez‐García *et al.*, 2014; Löwe *et al.*, 2017a,b, 2019; Akkaya *et al.*, 2018). During routine cultivation, *P. putida* strains carrying an integrated *dnt* gene cluster were maintained in the presence of 25 μg ml^−1^ gentamicin, while strains transformed with plasmid pSEVA221·*cscRABY* were maintained with 50 μg ml^−1^ kanamycin. Lysogeny broth (LB) medium cultures were at 32°C in a Multitron (Infors HT) incubator with rotary agitation at 150 rpm. For co‐culture experiments, *P. putida* cultures grown in LB medium were re‐suspended in M3 minimal medium (Table S1) with either 20 g l^−1^ or 2 g l^−1^ sucrose, as indicated in the main text. These cultures were then used as inoculum for subsequent experiments after pelleting cells, rinsing the pellet, and re‐suspending cells in fresh media to prevent the carryover of carbohydrates and other soluble metabolites. Antibiotic selection was omitted for all co‐culture experiments.

### **Encapsulation of *S*** *.* ***elongatus* CscB cells in alginate beads**

Alginate encapsulation was performed as previously described (Weiss *et al.*, 2017) with minor adjustments. Briefly, planktonic *S. elongatus* CscB cells grown in minimal BG‐11 medium containing 1 g l^−1^ HEPES (pH = 8.3; Sigma‐Aldrich Co., St. Louis, MO, USA) were harvested at an optical density at 750 nm (OD_750_) of 2.0 *via* centrifugation at 3500 *g* for 30 min and concentrated in 3 ml of sulfur‐free BG‐11 minimal medium. Concentrated cells were mixed into a sterile and degassed volume of 3% (w/v) sodium alginate at a final OD_750_ = 5.0, thereby resulting in a 2.75% (w/v) sodium alginate‐*S. elongatus* CscB suspension. In a sterile biosafety cabinet, a vertically oriented syringe pump (KD Scientific, Holliston, MA, USA) was used to dispense the solution dropwise through a 30 G needle into a ≥ 20‐fold larger volume of 20 mM BaCl_2_. The drops travelled ~ 35 cm from needle to the slowly stirred BaCl_2_ solution and were allowed to equilibrate in the solution for at least 20 min. Solidified beads were rinsed once with BG‐11 medium and incubated overnight in M3 minimal medium (without NaCl); the mean diameter of cured alginate beads used in these experiments was ~ 1.8 mm. To acclimate cells within the alginate hydrogel and precipitate residual Ba^2+^, beads were transferred through a series of media washes in the following days. The first day post‐encapsulation, beads were rinsed and re‐suspended in fresh M3 minimal medium (without NaCl), transferred to 250‐ml baffled Erlenmeyer flasks, and placed into a Multitron Pro incubator (Infors HT) with rotary shaking (125 rpm) for 24 h. Subsequently, the M3 minimal medium was exchanged, and the alginate bead suspension were portioned in ~ 10‐ml aliquots into 125‐ml baffled Erlenmeyer flasks. To acclimate cells to the final working salt concentrations, the spent medium was replaced with M3 minimal medium with only 25 mM NaCl for 24 h, then M3 minimal medium with 50 mM NaCl for 24 h, and finally to complete M3 minimal medium (containing 100 mM NaCl, Table S1). The culture medium was subsequently refreshed daily for 3–5 days prior to initiation of experiments. In the cases where the beads were utilized in co‐culture, *cscB* expression was induced with the addition of IPTG at 1 mM 24 h prior to the addition of *P. putida* cells. The culture medium was exchanged a final time coincident with addition of *P. putida* cells.

### Analytical methods

Culture optical densities were measured with a Genesys 20 (Thermo Fisher Scientific, Waltham, MA, USA) spectrophotometer. The density of planktonic *S. elongatus* CscB was measured at OD_750_, and both co‐culture experiments and *P. putida* mono‐cultures were measured at OD_600_. This spectrophotometer was also used to measure Chl(*a*) concentrations of planktonic *S. elongatus* cultures (Zavřel *et al.*, 2015). For Chl(*a*) measurements in alginate‐encapsulated *S. elongatus*, 1 ml of pre‐chilled methanol was added to four alginate beads in three technical replicates. Beads were vortexed and incubated at 4°C for 30 min in the dark to extract Chl(*a*). The absorbance of the supernatant was measured in cuvettes at 720 nm and 665 nm to calculate the final Chl(*a*) concentration as previously described (Zavřel *et al.*, 2015).

Sucrose concentration of beaded cultures was measured as indicated previously (Weiss *et al.*, 2017). Briefly, 1 ml of culture supernatant was withdrawn at the indicated time points, cells were pelleted at 17 000 *g* for 10 min, and sucrose was quantified *via* a sucrose/d‐glucose assay kit (Megazyme, Bray, Ireland) with three technical replicates for each sample. Culture supernatant spectra were scanned using a DU800 Spectrophotometer (Beckman Coulter Inc., Brea, CA, USA) to assess the presence of coloured metabolites within the 2,4‐DNT degradation pathway. Cell‐free culture supernatant was obtained by centrifugation for 10 min at 17 000 *g*. The supernatant was then transferred to a cuvette for spectral analysis.

Direct 2,4‐DNT measurements were made using an Agilent 5975 GC/single quadrupole MS (Agilent Technologies Inc., Santa Clara, CA, USA). Culture samples were centrifuged at 17 000 *g* for 10 min and 10 μl of cell‐free supernatant were removed to a new tube. A 50‐μl aliquot of ethyl acetate was added to the supernatant and incubated at room temperature for 30 min. The ethyl acetate phase was then transferred to a GC vial and injected (the injection volume was 1 μl). Samples were separated with a 5% (w/w) phenyl‐methyl capillary column (Agilent Technologies Inc.) and measured by mass selective detector (MSD). A temperature of 275°C was set for the split/splitless injector (ratio of 10:1). Helium gas was used as the carrier gas at a flow rate of 1 ml min^−1^. The release of inorganic nitrite (NO_2_^–^) from nitroaromatic compounds was assessed in culture supernatants by a colorimetric method essentially as described by Zhang *et al* *. * (2001).

LC‐MS measurements of metabolites within the 2,4‐DNT degradation pathway were made using a Waters Xevo G2‐XS UPLC‐MS/MS (Waters Corp., Milford, MA, USA). Culture samples were harvested and pelleted *via* centrifugation (17 000 *g* for 10 min), and a 100‐μl aliquot of the supernatant was transferred to a clean tube, lyophilized, and re‐suspended in 1 ml of deionized water for measurements. HPLC measurements of crotonic acid (as a proxy of PHA accumulation) were made with a Waters 2695 HPLC. Samples were processed and analysed as previously described (Weiss *et al.*, 2017). Briefly, cells were centrifuged at 17 000 *g* for 10 min, the supernatant was decanted, and the pellet was lyophilized. The cell biomass was suspended in 1 ml of concentrated H_2_SO_4_ and heated to 90°C for 1 h to promote dehydration of 3‐hydroxybutyrate into crotonic acid. After cooling the reaction mixture to room temperature, particulate material was removed by filtration and the mixture was diluted 100‐fold with deionized water. A 20‐μl aliquot of the resulting solution was injected onto an Aminex 300‐mm HPX‐87H column (Bio‐Rad Laboratories, Hercules, CA, USA), and 0.028 N H_2_SO_4_ was used as the mobile phase at a flow of 1 ml min^−1^. The column temperature was maintained at 60°C and the UV absorption of crotonic acid was monitored at 210 nm. Two standards of commercial PHA (Sigma‐Aldrich Co.) were similarly treated and used for quantification purposes.

A simple mass‐action model was used to explore the parameter space that could sufficiently fit our kinetic data sets of 2,4‐DNT transformation (Fig. 4 and Fig. S5; Voit, 2000, 2017). Briefly, metabolite flux through both reductive and oxidative branches was modelled for the initial steps of 2,4‐DNT transformation as a first‐order kinetic dependent upon the concentrate of each substrate (d[P]/dt = k_1_[S] – k_2_[P]); where P = product, S = substrate, and k_1_ and k_2_ are coefficients related to each branch point within the metabolic network. Reactions were modelled to be irreversible based on the unidirectionality of the early steps of 2,4‐DNT transformation. Models were capable of producing simulated metabolite profiles that fit our kinetic profiles (within an allowed 25% error of our experimentally measured values), including the loss of 2,4‐DNT from solution, and gain of the colorimetric products 4M5NC and 2H5MQ across a range of parameter space without making further assumptions of more complex pathway regulation (e.g. feedback or feed‐forward regulatory steps), including oxidative branch flux between 15 and 40% of the competing reductive pathway.

## Conflict of interest

None declared.

## Author contributions

D.T.F., P.I.N. and D.C.D. designed and directed the project and experiments. P.C. designed and engineered the *P. putida* strains utilized in this work. D.T.F. and P.S. performed the experiments and recorded the results. D.T.F., P.S., P.C., P.I.N. and D.C.D. analysed and interpreted the data. D.T.F. drafted the manuscript and figures. D.T.F., P.S., P.C., P.I.N. and D.C.D. provided commentary and edits to the manuscript and figures and prepared the final version of the article.

## Supporting information


**Table S1**. Chemical composition of BG‐11, M3, and M3‐N minimal media used in this study.
**Fig. S1**. Influence of 2,4‐DNT on growth of *S. elongatus *PCC 7942.
**Fig. S2**. Effect of 2,4‐DNT on alginate‐encapsulated *S. elongatus *CscB.
**Fig. S3**. Chlorophyll *a *content per cell comparison in planktonic and alginate‐encapsulated *S. elongatus *CscB.
**Fig. S4**. Spectral analysis of supernatants of *P. putida *EM·S cultures.
**Fig. S5**. Kinetics of 2,4‐DNT transformation by engineered *P. putida *through the oxidative and reductive pathway.
