# Supplementary material for: Biotransformation of 2,4‐dinitrotoluene in a phototrophic co‐culture of engineered Synechococcus elongatus and Pseudomonas putida
Source: Microb Biotechnol. 2020 Feb 16;13(4):997–1011. doi: 10.1111/1751-7915.13544 (PMC7264894; doi:10.1111/1751-7915.13544)
Supplement: Supplementary file 1 — Table S1. Chemical composition of BG‐11, M3, and M3‐N minimal media used in this study. Fig. S1. Influence of 2,4‐DNT on growth of S. elongatus PCC 7942. Fig. S2. Effect of 2,4‐DNT on alginate‐encapsulated S. elongatus CscB. Fig. S3. Chlorophyll a content per cell comparison in planktonic and alginate‐encapsulated S. elongatus CscB. Fig. S4. Spectral analysis of supernatants of P. putida EM·S cultures. Fig. S5. Kinetics of 2,4‐DNT transformation by engineered P. putida through the oxidative and reductive pathway. [file MBT2-13-997-s001.pdf]

Biotransformation of 2,4-dinitrotoluene in a phototrophic co-culture of engineered *Synechococcus elongatus* and *Pseudomonas putida*

Derek T. Fedeson[1,2], Pia Saake[3], Patricia Calero[4], Pablo Iván Nikel[4] & Daniel C. Ducat[1,2,5]

[1] DOE-MSU Plant Research Laboratories, Michigan State University, East Lansing, Michigan, USA.

[2] Genetics Program, Michigan State University, East Lansing, Michigan, USA.

[3] Heinrich-Heine Universität, Düsseldorf, Germany.

[4] The Novo Nordisk Foundation Center for Biosustainability, Technical University of Denmark, Kgs Lyngby, Denmark.

[5] Department of Biochemistry and Molecular Biology, Michigan State University, East Lansing, Michigan, USA.

Table S1. Chemical composition of BG-11, M3, and M3-N minimal media used in this study.

Chemical Name	Chemical formula	Concentration (M) in medium		
		BG-11	M3	M3-N
Ammonium iron(III) citrate	$(NH_4)_5[Fe(C_6H_4O_7)_2]$	2.29×10^{-5}	2.29×10^{-5}	2.29×10^{-5}
Calcium chloride dihydrate	$CaCl_2\cdot2H_2O$	2.45×10^{-4}	2.45×10^{-4}	2.45×10^{-4}
Citric acid	$C_6H_8O_7$	3.12×10^{-5}	3.12×10^{-5}	3.12×10^{-5}
Cobalt(II) nitrate hexahydrate	$Co(NO_3)_2\cdot6H_2O$	1.7×10^{-7}	1.7×10^{-7}	1.7×10^{-7}
Copper(II) sulfate pentahydrate	$CuSO_4\cdot5H_2O$	3.16×10^{-7}	3.16×10^{-7}	3.16×10^{-7}
Dibasic potassium phosphate	K_2HPO_4	2.3×10^{-4}	4.73×10^{-3}	4.73×10^{-3}
Dihydrogen borate	H_3BO_3	4.7×10^{-5}	4.7×10^{-5}	4.70×10^{-5}
Ethylenediaminetetraacetic acid (EDTA)	$C_{10}H_{16}N_2O_8Na_2$	3.42×10^{-6}	3.42×10^{-6}	3.42×10^{-6}
Magnesium sulfate heptahydrate	$MgSO_4\cdot7H_2O$	3.04×10^{-4}	3.04×10^{-4}	3.04×10^{-4}
Manganese(II) chloride hydrate	$MnCl_2\cdot4H_2O$	9.15×10^{-6}	9.15×10^{-6}	9.15×10^{-6}
Sodium carbonate	Na_2CO_3	1.89×10^{-4}	1.89×10^{-4}	1.89×10^{-4}
Sodium molybdate dihydrate	$Na_2MoO_4\cdot2H_2O$	1.61×10^{-6}	1.61×10^{-6}	1.61×10^{-6}
Sodium nitrate	$NaNO_3$	1.76×10^{-2}	1.76×10^{-2}	2×10^{-3}
Zinc sulfate heptahydrate	$ZnSO_4\cdot7H_2O$	7.72×10^{-7}	7.72×10^{-7}	7.72×10^{-7}
Ammonium chloride	NH_4Cl	—	4×10^{-3}	—
Sodium chloride	NaCl	—	0.1	0.1

Figure S1. Influence of 2,4-DNT on growth of *S. elongatus* PCC 7942.

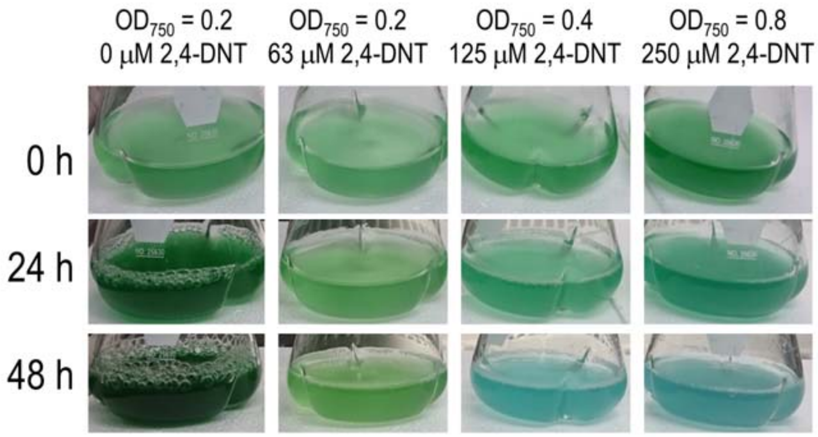

Visual representation of the impact of varying concentrations of 2,4-DNT (up to 250 μM) on the growth of wild-type *S. elongatus* PCC 7942 over 48 h. At higher initial inocula of *S. elongatus*, 2,4-DNT can still result in growth impairment. Cell growth was estimated as the optical density measured at 750 nm (OD_{750}).

Figure S2. Effect of 2,4-DNT on alginate-encapsulated *S. elongatus* CscB.

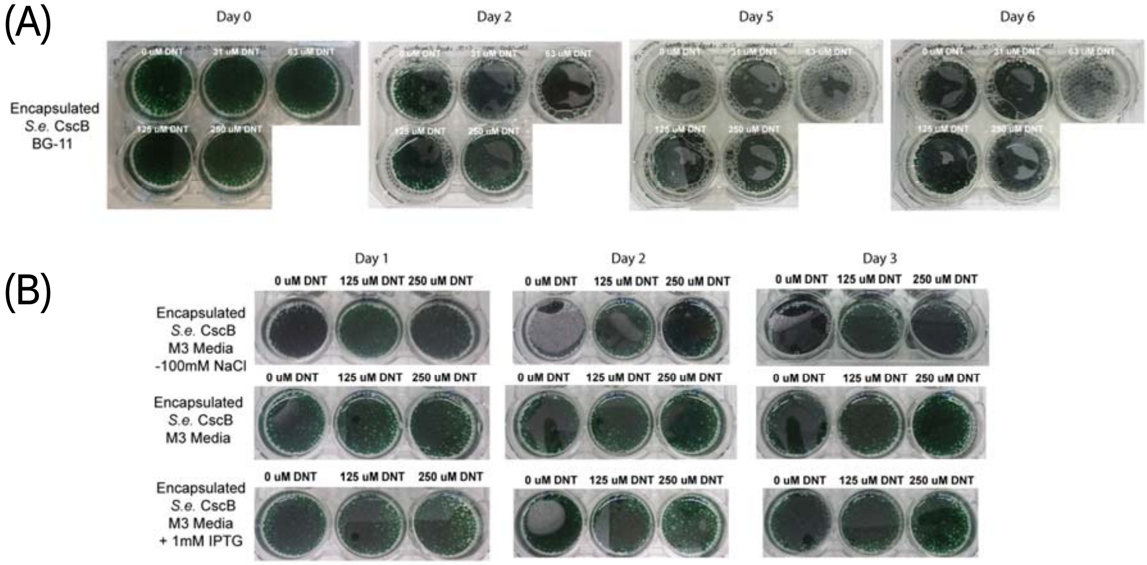

(A) Cultures of alginate-encapsulated *S. elongatus* CscB cells incubated in BG-11 minimal medium with different added concentrations of 2,4-DNT [0, 125 and 250 μM] were followed over the course of 6 days. In contrast to the planktonic cultures of *S. elongatus* PCC 7942, these cultures did not display the same bluing and bleaching transition associated with the toxic effects of the 2,4-DNT. (B) Encapsulated *S. elongatus* CscB cells were tested with this same range of 2,4-DNT concentrations in M3 minimal medium modified for co-culture (without 100 mM NaCl) and in M3 minimal medium with or without 1 mM IPTG (in the presence of IPTG, the system is in a fully induced condition that allows for the accumulation and export of sucrose into the culture supernatant). This last condition (i.e., with added IPTG) is the one utilized for all co-cultures described in this study unless otherwise stated.

Figure S3. Chlorophyll *a* content per cell comparison in planktonic and alginate-encapsulated *S. elongatus* CscB.

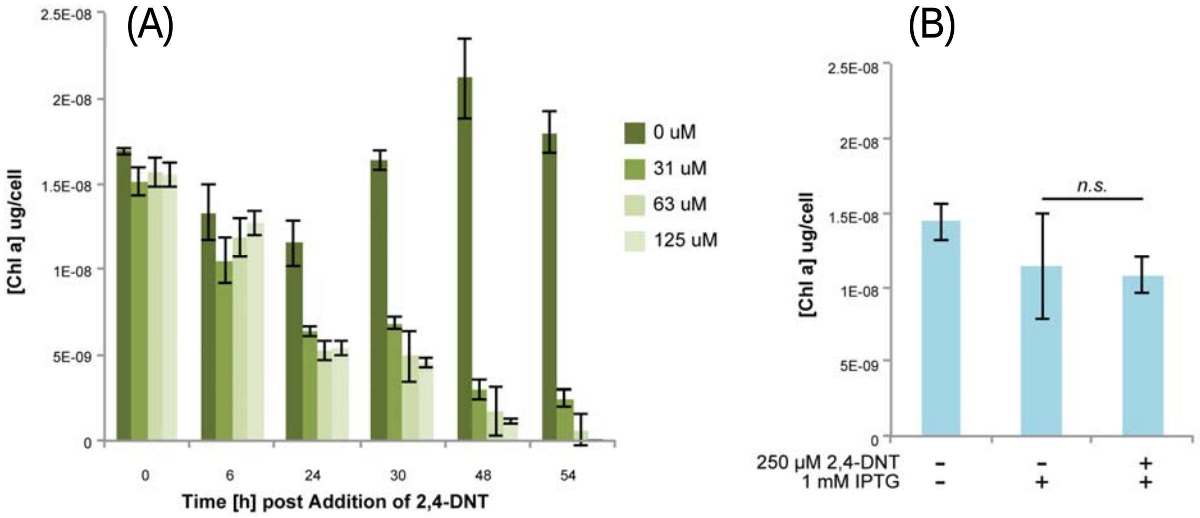

(A) Per-cell chlorophyll *a* [Chl(*a*)] content of planktonic *S. elongatus* CscB cells when exposed to a range of concentrations of 2,4-DNT—as in the experiment displayed in Fig. 2B in the main text. All cultures that have been added with 2,4-DNT exhibited a steady decline in the Chl(*a*) content per cell. (B) Per-cell Chl(*a*) content of encapsulated *S. elongatus* CscB—as in the experiment displayed in Fig. 2C in the main text. To calculate the Chl(*a*) concentration per cell for alginate-encapsulated cyanobacteria, it was necessary to estimate the starting concentration of encapsulated *S*. *elongatus* CscB cells. With an estimated cell density of 3.3×10^8 *S. elongatus* PCC 7942 cells per OD_{750} per 1 mL, and a bead volume based on the measured diameter of alginate beads (~2.6 mm), we were able to calculate that each bead should have approximately 1.52×10^7 cyanobacteria cells upon formation. As we have previously shown that *S*. *elongatus* CscB encapsulated within Ba^{2+}-alginate hydrogels are extremely restricted in division (with a doubling time >> weeks), we assumed that this initial cell count remained essentially unchanged for the purposes of these estimates. n.s., difference not significant.

Figure S4. Spectral analysis of supernatants of *P. putida* EM·S cultures.

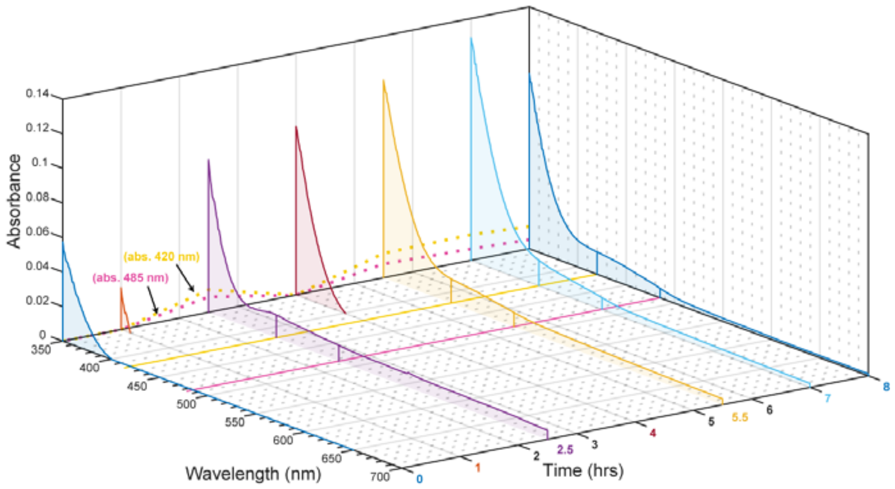

Time-course of averaged spectral signatures (*n* = 3) in supernatants from *P. putida* EM·S cultures grown in M3 minimal medium in the presence of 250 μM 2,4-DNT, as measured *via* scanning spectrophotometry. The signature absorbance peaks of 4M5NC and 2H5MQ are indicated with a yellow and purple line, respectively. Abs., absorbance at the indicated wavelength.

Figure S5. Kinetics of 2,4-DNT transformation by engineered *P. putida* through the oxidative and reductive pathway.

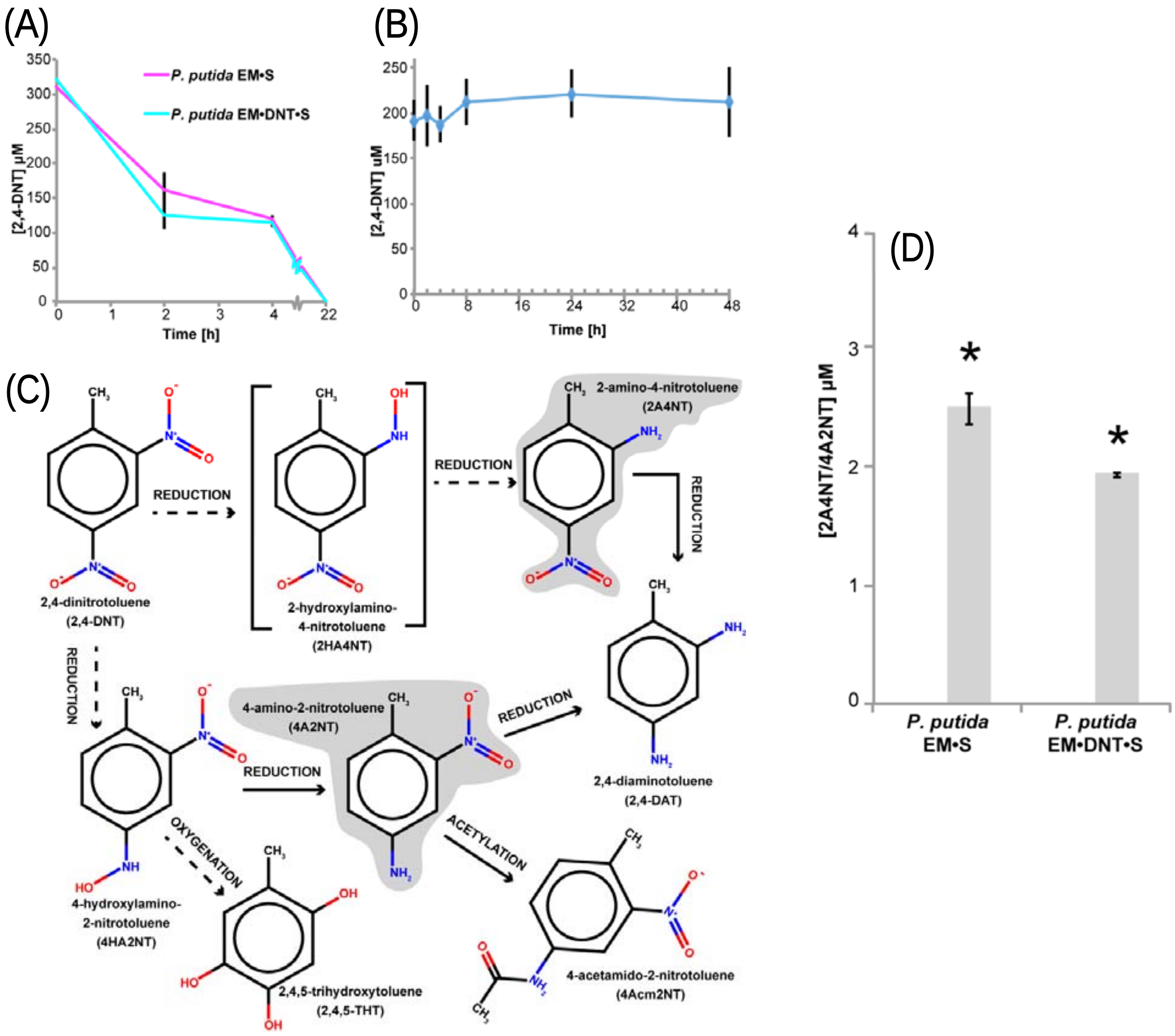

(A) Time-course of the concentration of 2,4-DNT in cultures of engineered *P. putida* grown in M3 minimal medium with 20 g/L sucrose and 250 µM 2,4-DNT as measured by GC-MS. (B) Control experiments, in which 2,4-DNT was added to M3 minimal medium in the absence of biomass. These control experiments were incubated under the same conditions indicated for panel (A). In all cases, there were no apparent changes in the concentration of 2,4-DNT over 48 h as assessed by GC-MS. (C) Schematic of the reductive degradation pathway for 2,4-DNT in bacteria, indicating the biochemical transformations therein. Molecules in brackets are hypothetical. (D) LC-MS quantification of 2-amino-4-nitrotoluene/4-amino-2-nitrotoluene (2A4NT/4A2NT, intermediates of the reductive pathway) in culture supernatants sampled at 4 h. Statistical analysis showed that this decrease in the concentration of 2A4NT/4A2NT was significant (*P*-value = 0.02568) when comparing cultures of *P. putida* EM·S and *P. putida* EM·DNT·S. In all cases, the average values of biological replicates are reported and the error bars represent standard deviations from n = 3.
